# Supplementary material for: Self-driven electrical triggering system activates tunneling nanotube highways to enhance drug delivery in bladder cancer therapy
Source: Nat Commun. 2025 Nov 18;16:10093. doi: 10.1038/s41467-025-65017-2 (PMC12627709; doi:10.1038/s41467-025-65017-2)
Supplement: Supplementary file 1 — Supplementary Information [file 41467_2025_65017_MOESM1_ESM.docx]

Supporting Information

**Self-driven Electrical Triggering System Activates Tunnel Nanotube Highways to Enhance Drug Delivery in Bladder Cancer Therapy**

Zhijun Liu^1,2^, Ravindra Joshi^3,4^, Zhongguo Zhou^5^, Fulin Liu^6,7^, Ying Gong^1^, Mingyan Sun^1^, Xiuxiu Li^1^, Tao Jiang^1^, Liang Zou^8^, Siyuan Wang^9^, Yi Shi^6,7^*, Zong-Hong Lin^3,4^*, Yang-Bao Miao^1^*

^1^ Z. Liu, Y. Gong, M. Sun, X. Li, T. Jiang, Prof. Y. Shi, Y. Miao

Department of Haematology, Sichuan Academy of Medical Sciences & Sichuan Provincial People’s Hospital, School of Medicine of University of Electronic Science and Technology of China, Chengdu 610072, China.

E-mail: [miaoyangbao@uestc.edu.cn](mailto:miaoyangbao@uestc.edu.cn), [shiyi1614@126.com](mailto:shiyi1614@126.com),

^2^ Z. Liu

Department of Urology, South China Hospital, Medical School, Shenzhen University, Shenzhen 518116, China. National-Regional Key Technology Engineering Laboratory for Medical Ultrasound, School of Biomedical Engineering, Shenzhen University Medical school, Shenzhen 518060, China

^3^ Ravindra Joshi, Prof. Z. H. Lin

Department of Biomedical Engineering, National Taiwan University, Taipei 10167, Taiwan.

E-mail: [zhlin@ntu.edu.tw](mailto:zhlin@ntu.edu.tw)

^4^ Ravindra Joshi, Prof. Z. H. Lin

Department of Power Mechanical Engineering, National Tsing Hua University, Hsinchu 30013, Taiwan.

E-mail: [zhlin@ntu.edu.tw](mailto:zhlin@ntu.edu.tw)

^5^ Z. Zhou

Department of Surgery & Cancer, Faculty of Medicine, Imperial College London, London, UK

^6^ Dr. F. Liu, Prof. Y. Shi

Sichuan Provincial Key Laboratory for Human Disease Gene Study and the Center for Medical Genetics, Department of Laboratory Medicine, Sichuan Academy of Medical Sciences and Sichuan Provincial People's Hospital, University of Electronic Science and Technology of China, Chengdu 610072, China.

E-mail: [shiyi1614@126.com](mailto:shiyi1614@126.com)

^7^ Dr. F. Liu, Prof. Y. Shi

Research Unit for Blindness Prevention of Chinese Academy of Medical Sciences (2019RU026), Sichuan Academy of Medical Sciences and Sichuan Provincial People's Hospital, Chengdu 610072, China.

E-mail: [shiyi1614@126.com](mailto:shiyi1614@126.com)

^8^ Prof. L. Zou

School of Food and Biological Engineering, Chengdu University, Chengdu 610106, China.

^9^ S. Wang

Urology (Department), Sichuan Clinical Research Center for Cancer, Sichuan. Cancer Hospital & Institute, Sichuan Cancer Center, Affiliated Cancer Hospital of University of Electronic Science and Technology of China, Chengdu 610041, China.

**Supplementary Figs**


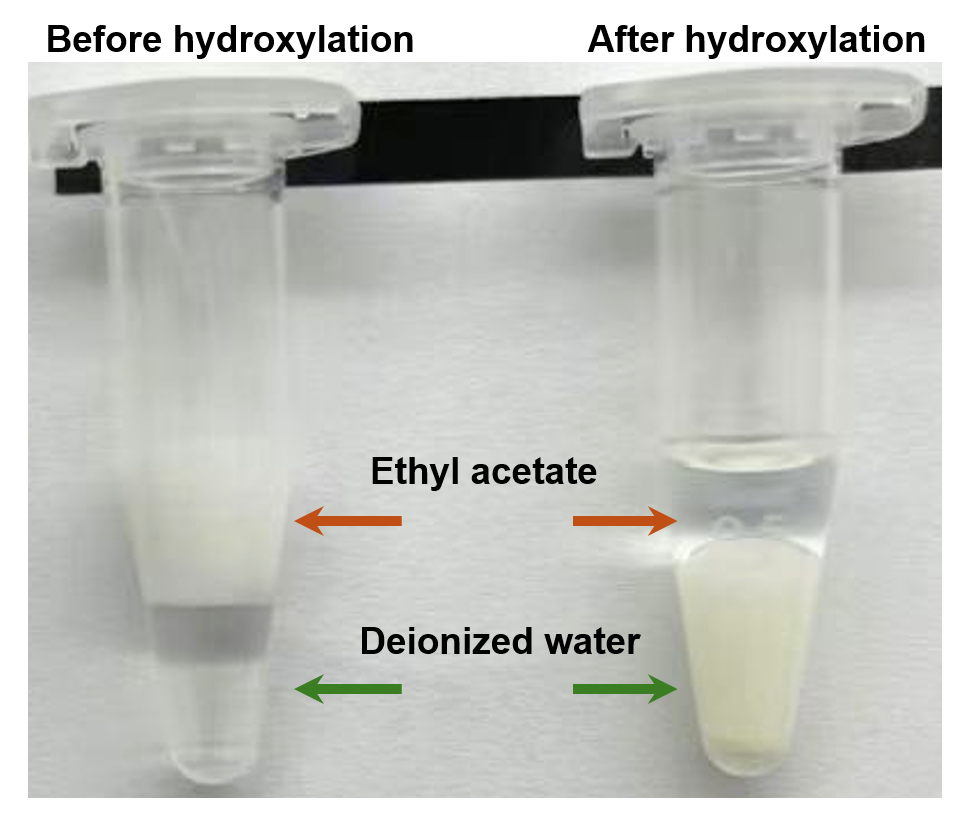


**Supplementary Fig. 1 The picture of BTO in different solution before and after surface hydroxylation.** The surface of BTO became hydrophilic after treatment with hydrogen peroxide solution for 8 h at 85 °C.


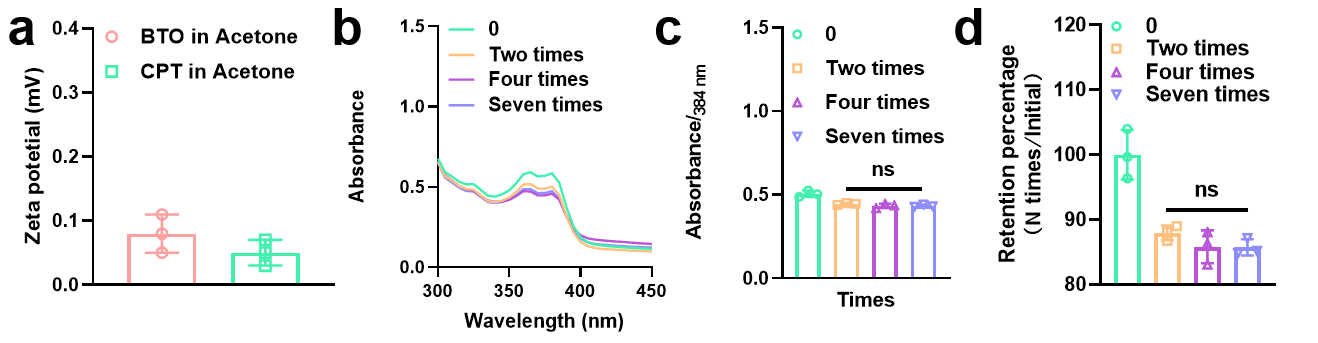


**Supplementary Fig. 2 Proof that CPT binds to BTO by chemical coupling. a)** ξ-potential of BTO nanoparticles and CPT in acetone (n = 3), **b)** ultraviolet-visible (UV-Vis) absorption spectra of the precipitate before and after washed, **c)** the absorbance value (λ= 384 nm) of the precipitate after washed several times (n = 3), **d)** the residue percentage of CPT after washed (n = 3).


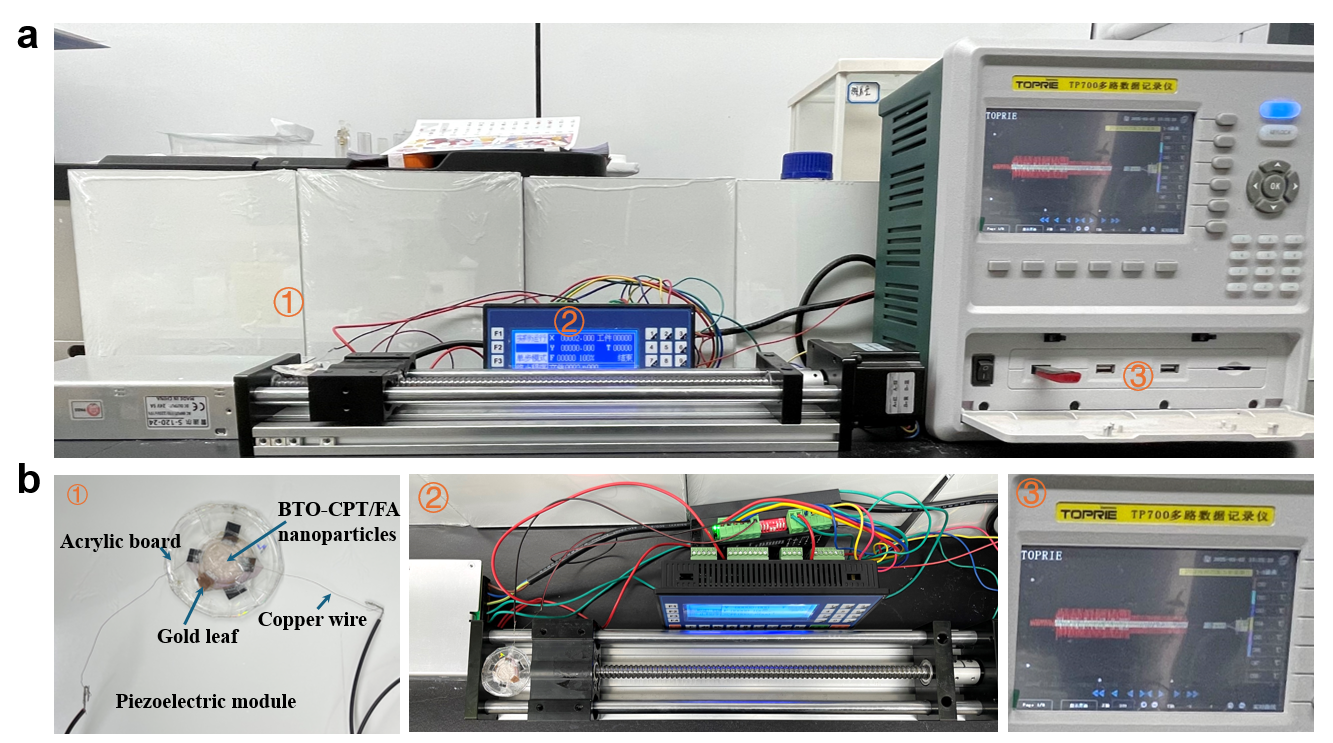


**Supplementary Fig. 3 Electrical signal measurement device. a)** The picture of the electrical signal measurement device, **b)** The composition of the electrical signal measurement device: piezoelectric module (①), linear reciprocating motion meter (②), and electrical signal detection workstation (③).


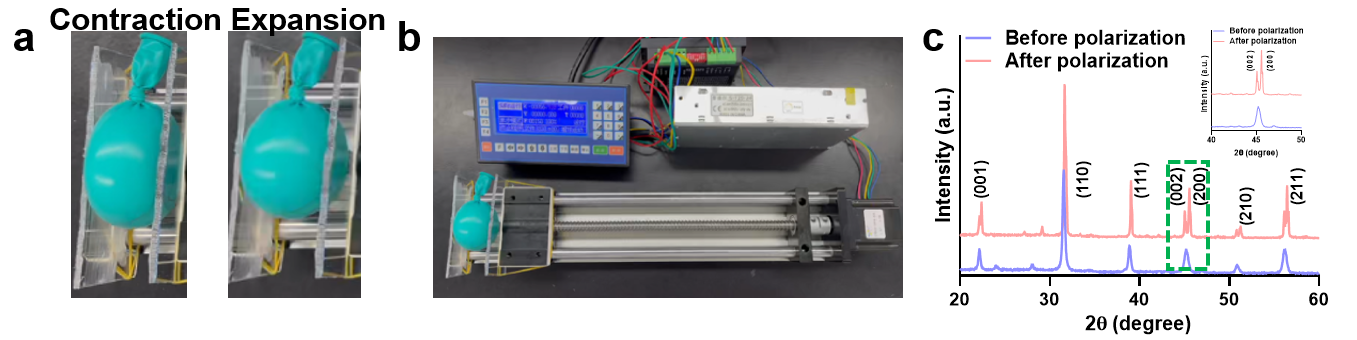


**Supplementary Fig. 4 Evidence for drug release based on electrical triggering.** A device for monitoring the drug release rely on electrical stimulation, a) The water balloon is in a state of contraction to simulate bladder contraction (left); the water balloon is in a state of expansion to simulate bladder expansion (right). b) The device includes a water balloon filled with the BTO-CPT/FA nano-system (30 ml), a linear reciprocating motion instrument, and double-layer acrylic sheet (with holes). c) X-ray diffraction (XRD) patterns of BTO-CPT/FA before and after polarization.


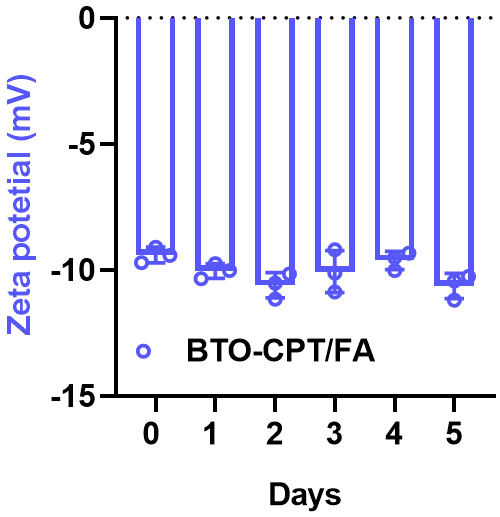


**Supplementary Fig. 5** The ξ-potential of BTO-CPT/FA within five days in artificial urine.


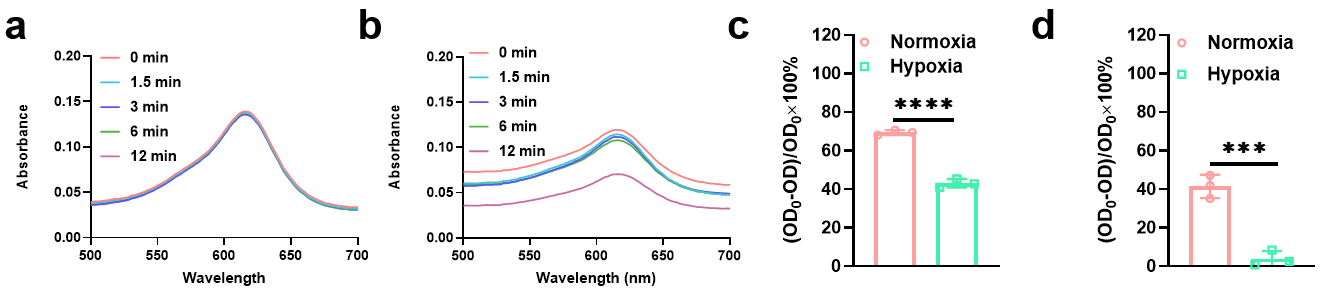


**Supplementary Fig. 6 Effect of dissolved oxygen in deionized water on the degradation of malachite green. a)** UV-vis absorption curve of malachite green after treated by control group under ultrasound in deionized water for oxygen removal, **b)** UV-vis absorption curve of malachite green after treated with BTO-CPT/FA group under ultrasound in deionized water for oxygen removal, **c)** Degradation of malachite green after treated by control group under ultrasound before and after oxygen removal (n = 3), **d)** Degradation of malachite green after treated with BTO-CPT/FA under ultrasound before and after oxygen removal (n = 3). ****p* < 0.001, *****p* < 0.0001.


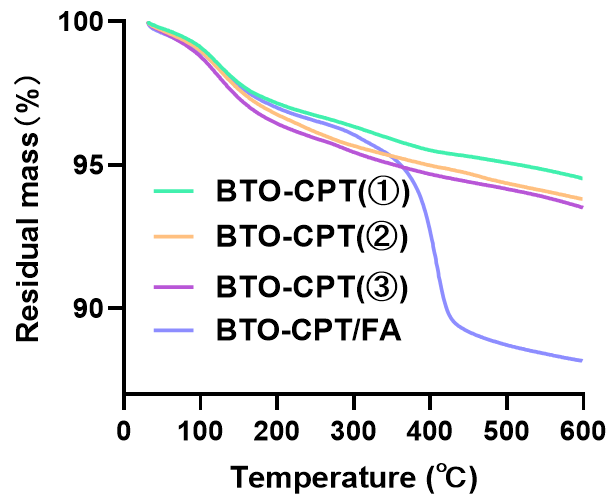


**Supplementary Fig. 7 The curve of thermogravimetric analysis (TGA).**


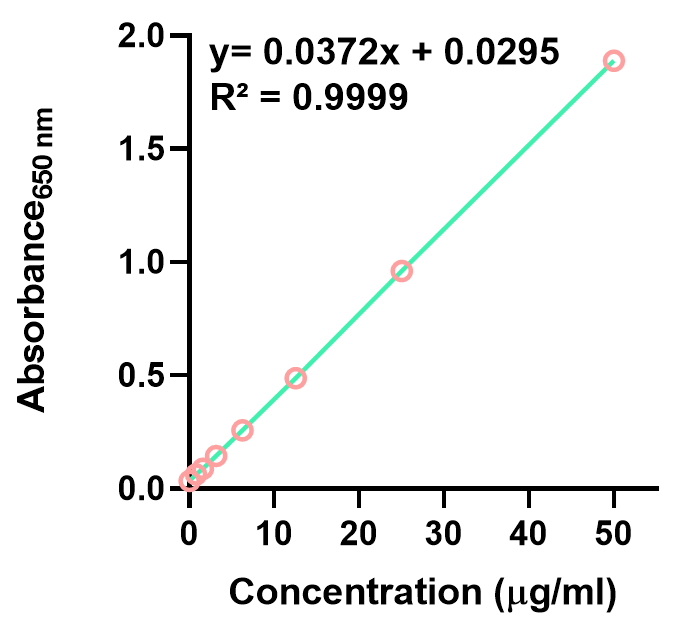


**Supplementary Fig. 8 Standard curve based on Cy5.5.**


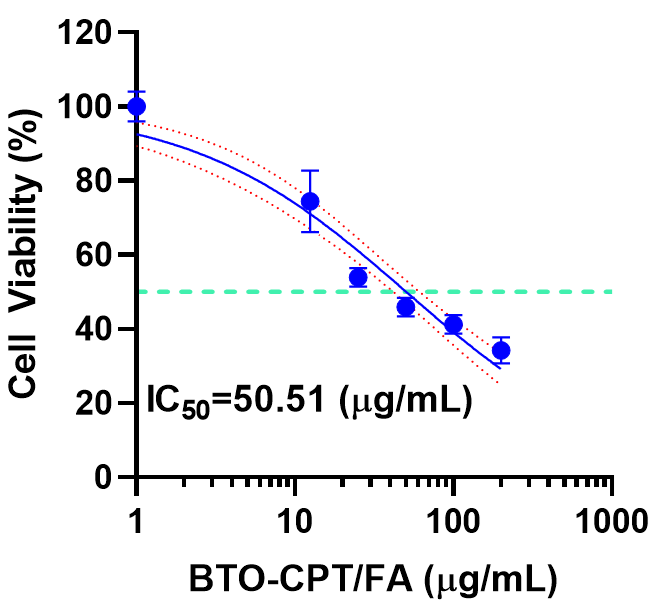


**Supplementary Fig. 9** The IC50 curve of BTO-CPT/FA. The red dashed line shows the confidence interval (95%).


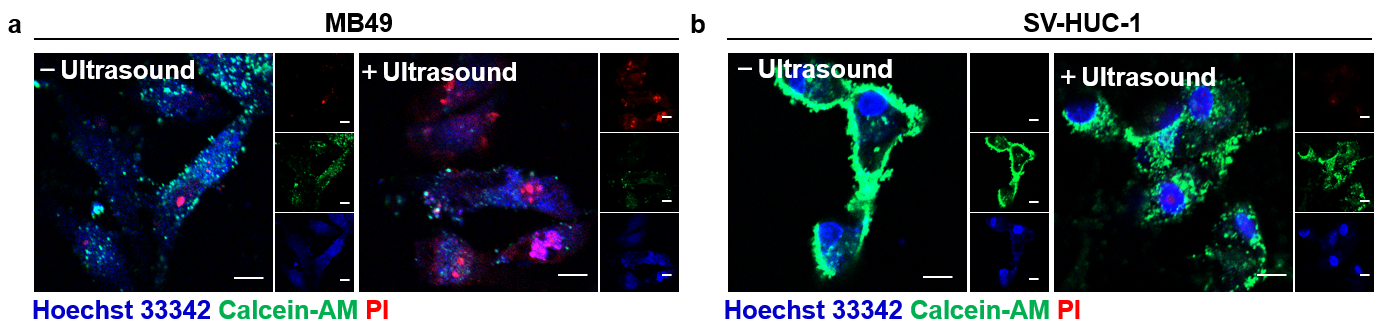


**Supplementary Fig. 10** Confocal images of live/dead cell staining for different cells after treated with different conditions, where green and red colors represent Calcein AM and PI fluorescence, respectively, scale bar = 10 µm.


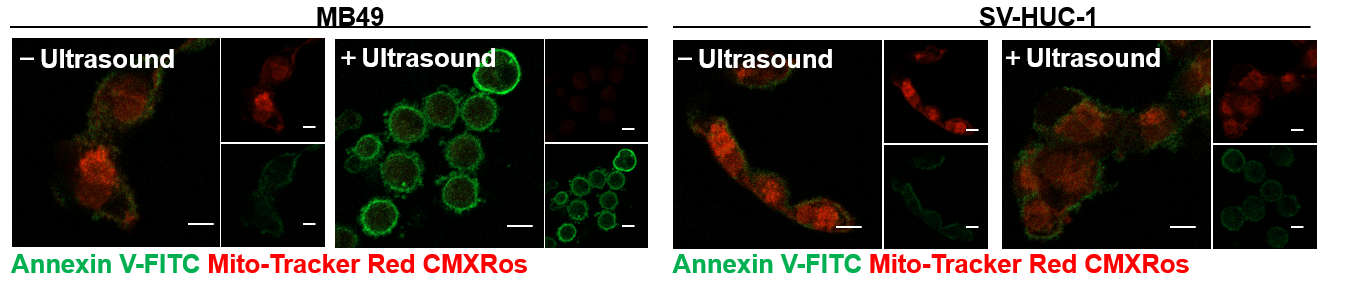


**Supplementary Fig. 11** Confocal images of membrane potential for different cells after treated with different conditions, where green and red colors represent Annexin V-FITC and Mito-Tracker Red CMXRos fluorescence, respectively, scale bar = 10 µm.


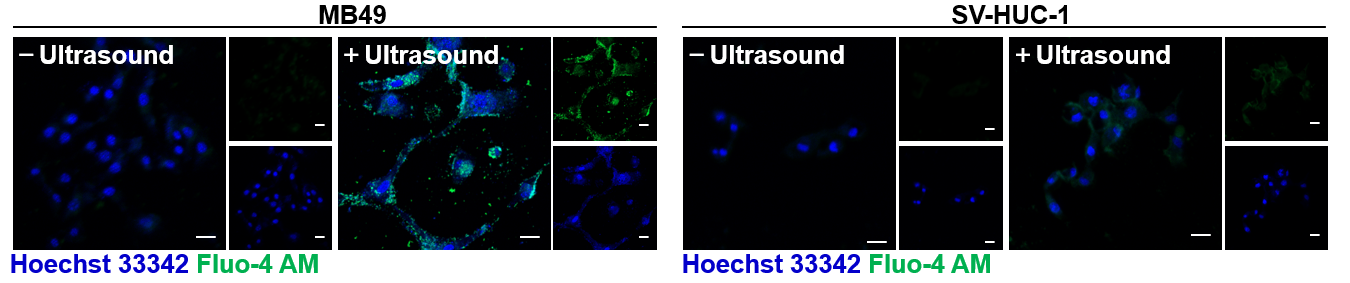


**Supplementary Fig. 12** Confocal images of calcium ion channels for different cells after treated with different conditions, where green and blue colors represent Fluo-4 AM and Hoechst 33342 fluorescence, respectively, scale bar = 20 µm.


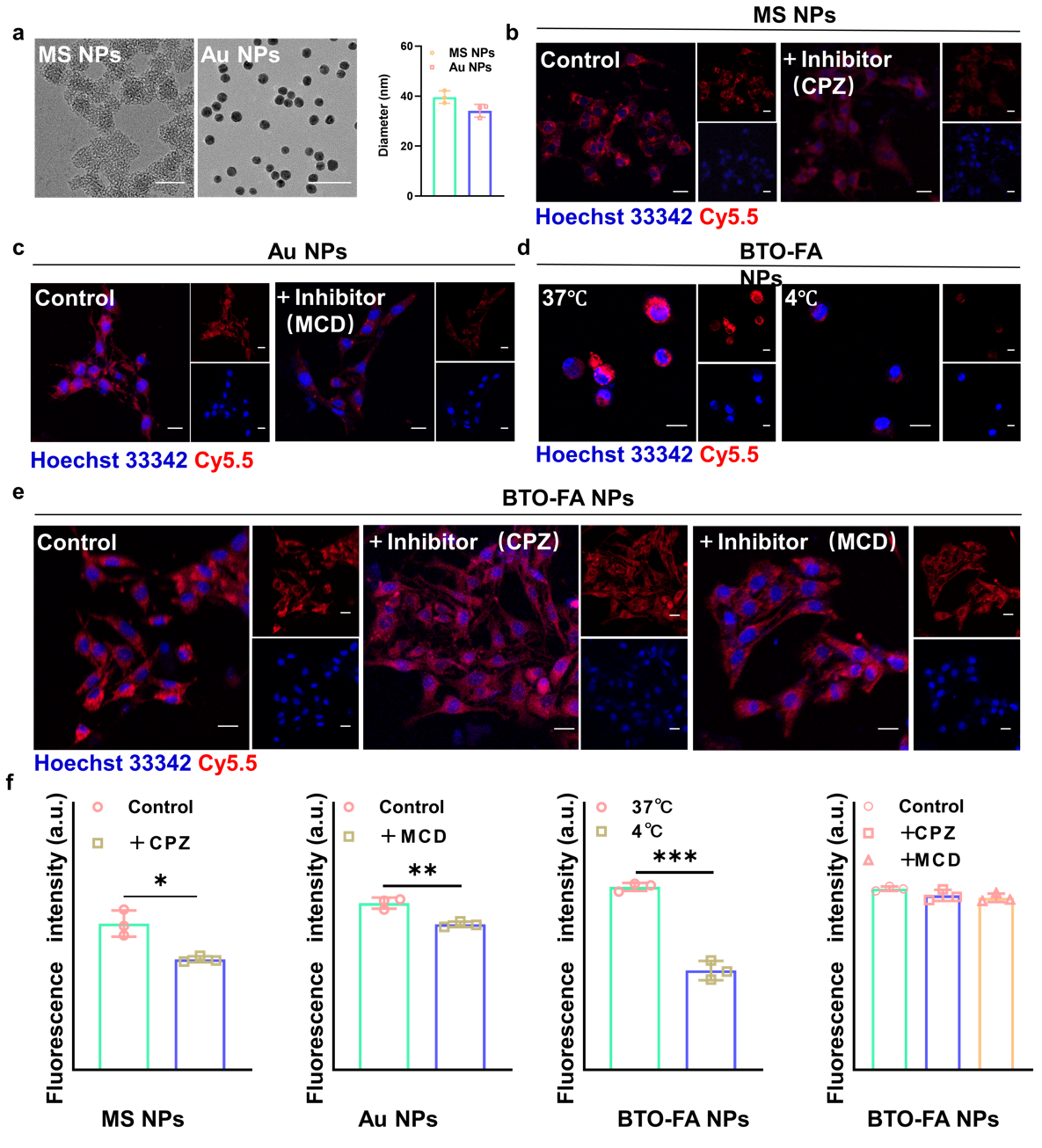


**Supplementary Fig. 13** Investigation of endocytosis mechanism. a) TEM images of MS NPs and Au NPs, scale bar = 50 nm; b) Confocal images of MS NPs after pretreated with CPZ, scale bar = 20 µm; c) Confocal images of Au NPs after pretreated with MCD, scale bar = 20 µm; d) Confocal images of BTO-FA NPs after pretreated with different temperature, scale bar = 10 µm; e) Confocal images of BTO-FA NPs after pretreated with different inhibitors, scale bar = 20 µm; where red and blue colors represent Cy5.5 and Hoechst 33342 fluorescence, respectively. f) Semi-quantitative analysis of fluorescence under different experimental conditions (n = 3). **p* = 0.0101, ***p* = 0.0042, ****p* = 0.0002.


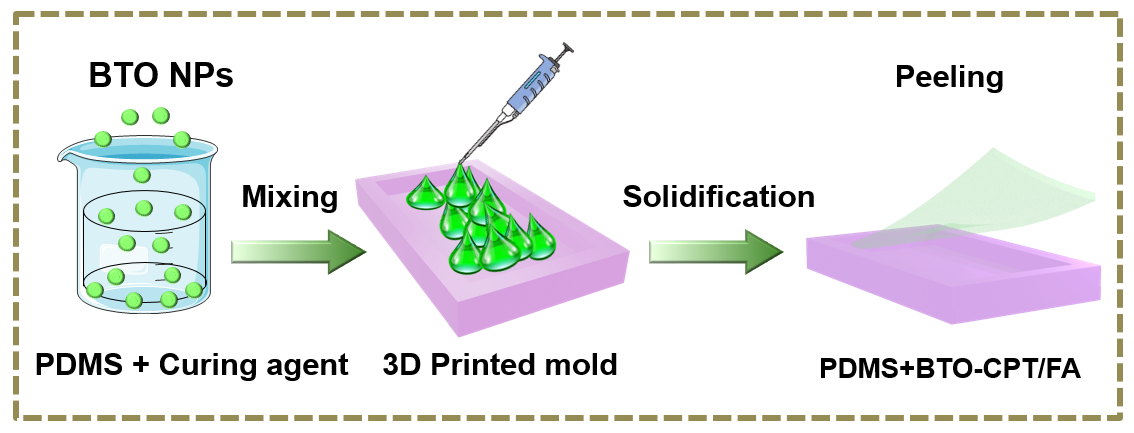


**Supplementary Fig. 14 Schematic illustration of the preparation of a PDMS piezoelectric patch cured with BTO-CPT/FA nanoparticles.** BTO-CPT/FA nanoparticles were mixed with a solvent containing PDMS and curing agent and cured to obtain a PDMS piezoelectric layer contained BTO-CPT/FA. Figure created in Blender 4.2. (https://www.blender.org/).


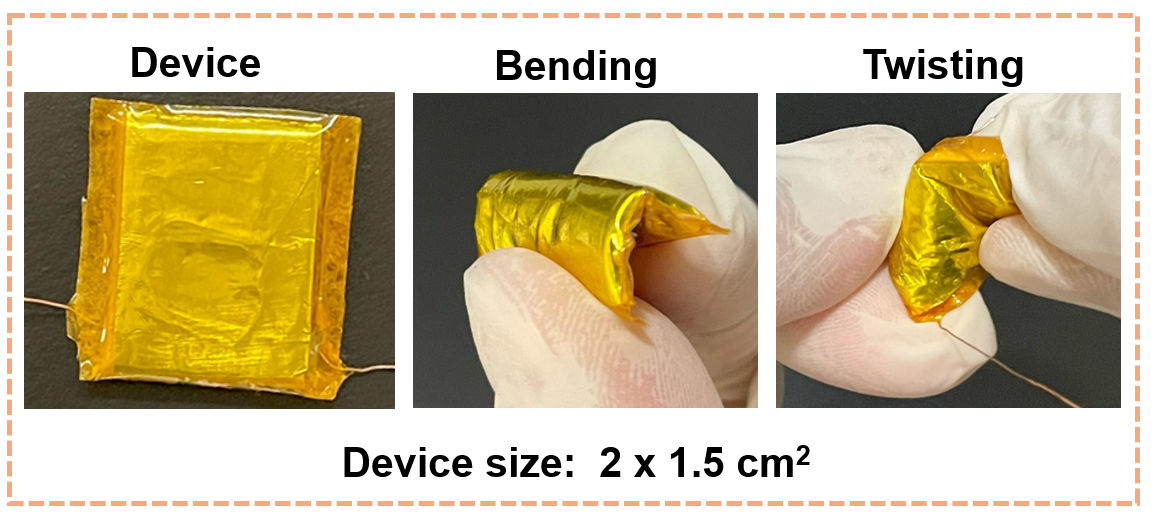


**Supplementary Fig. 15 Schematic illustration of the preparation of a PDMS piezoelectric patch cured with BTO-CPT/FA nanoparticles.** The pictures of the structure of the PDMS piezoelectric layer solidified with BTO-CPT/FA nanoparticles, when the piezoelectric layer stayed in normal, bended and twisted, respectively.


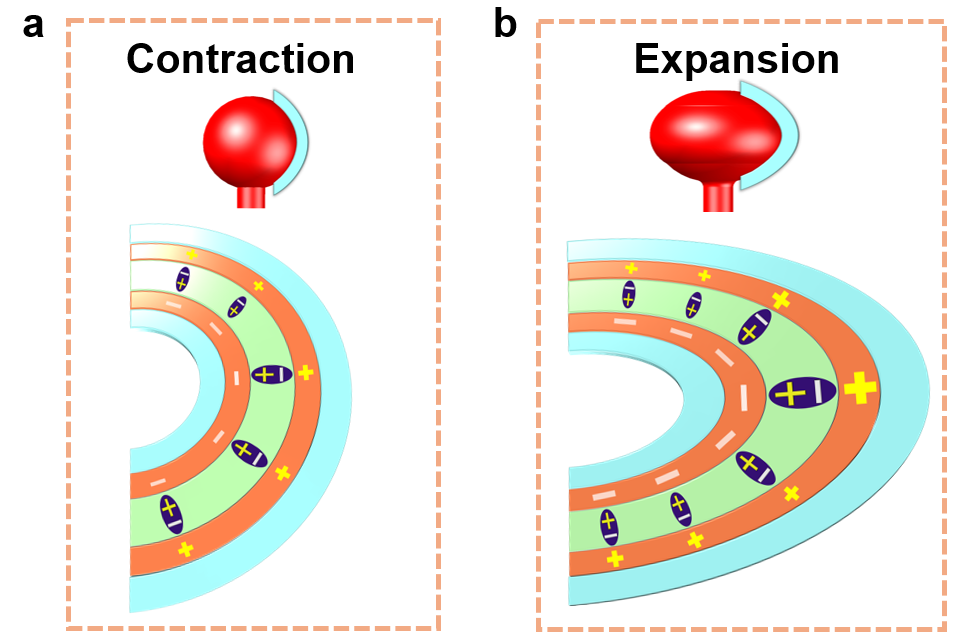


**Supplementary Fig. 16 Illustration of electric charge distribution when the simulated bladder was in contraction and** **expansion, respectively.** Figure created in AutoCAD 2024. (https://www.autodesk.com/in/products/autocad/overview).


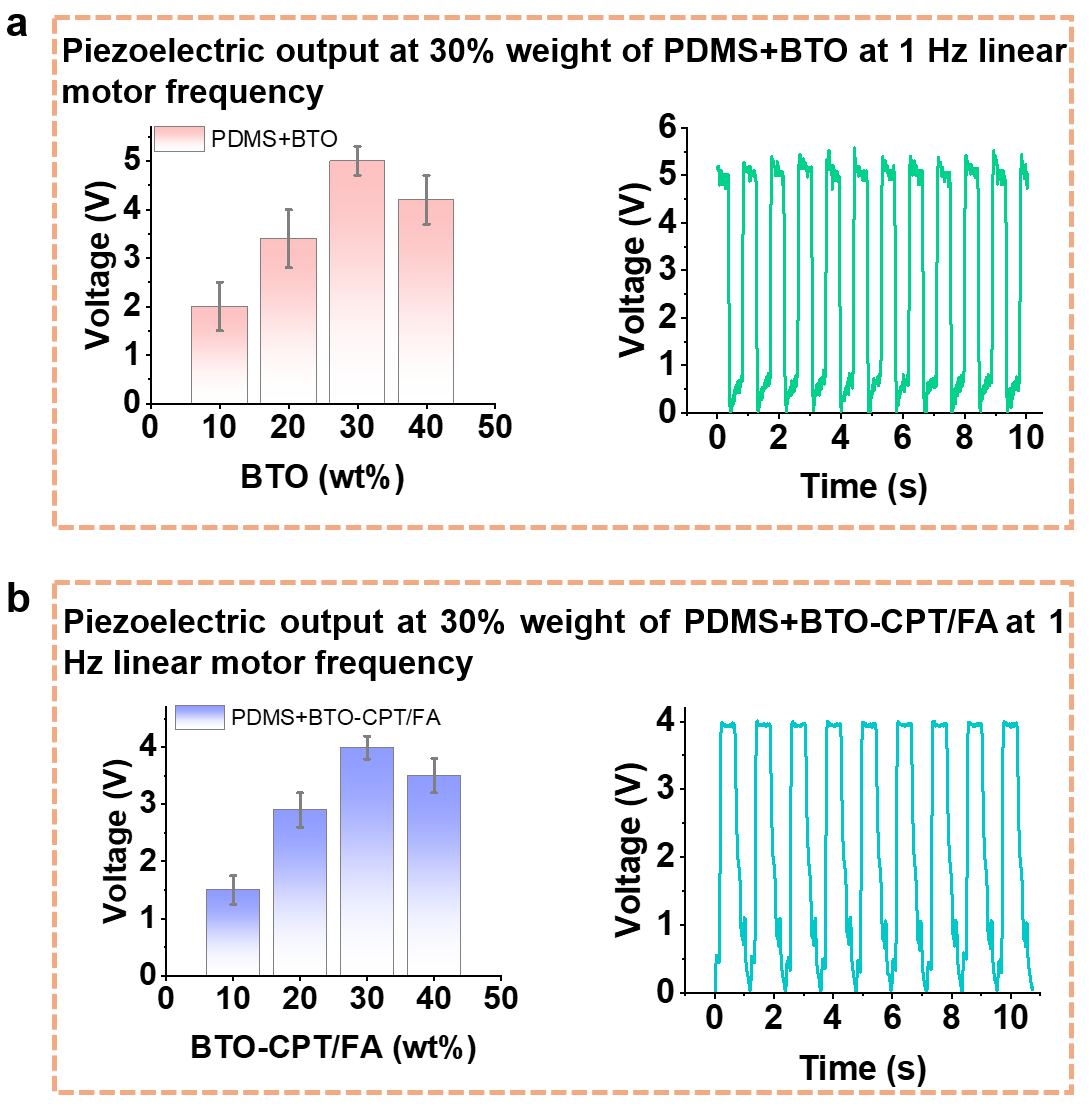


**Supplementary Fig. 17 The electromechanical performance of PENG device was further analyzed using device with dimensions (20 × 15 × 0.7 mm³) at a driving frequency of 1 Hz. a)** Output voltage of PDMS + BTO NPs piezoelectric layer (left) and the optimization of weight % BTO NPs (right). **b)** Output voltage of PDMS + BTO-CPT/FA NPs piezoelectric layer (left) and the optimization of weight % BTO-CPT/FA NPs (right).


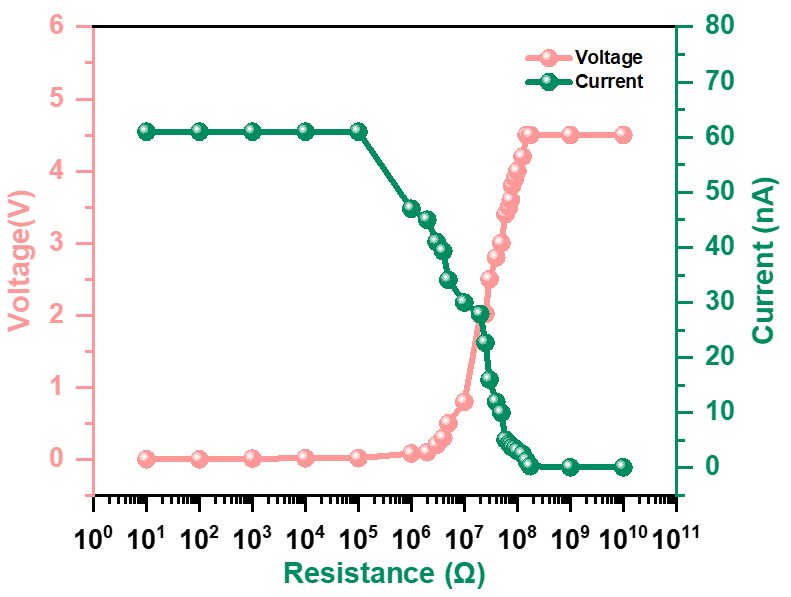


**Supplementary Fig. 18 Voltage-current curves of BTO-PDMS piezoelectric layer after bending-stretching.**


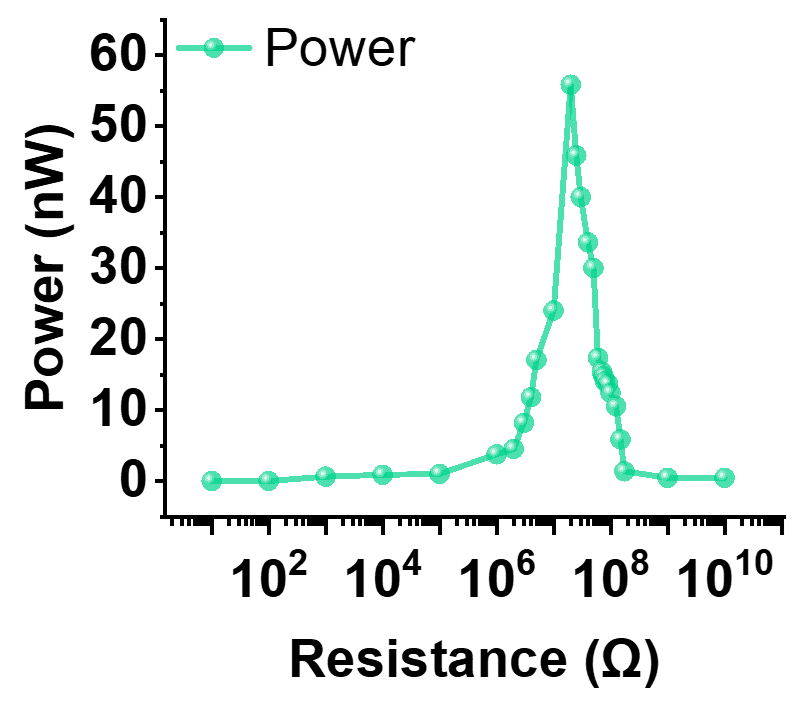


**Supplementary Fig. 19 Acting curve of BTO-PDMS piezoelectric layer after bending-stretching.**


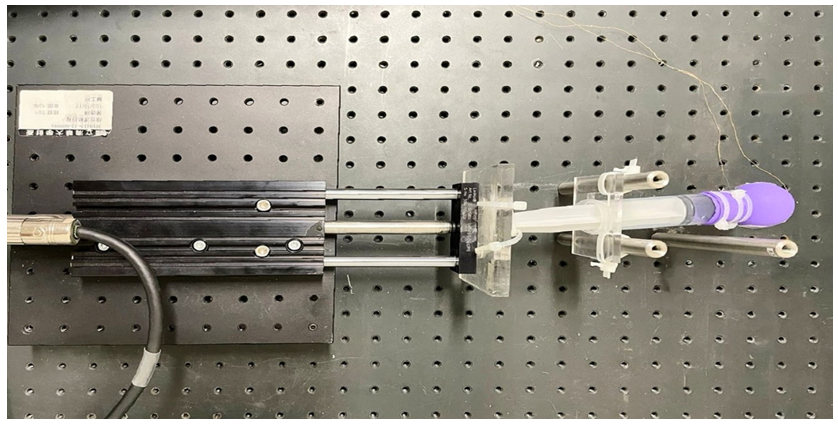


**Supplementary Fig. 20 The picture of an experimental setup for replicate the bladder behavior of storage (expansion) and voiding (contraction), its containing a rubber balloon and a 30 mL syringe.**


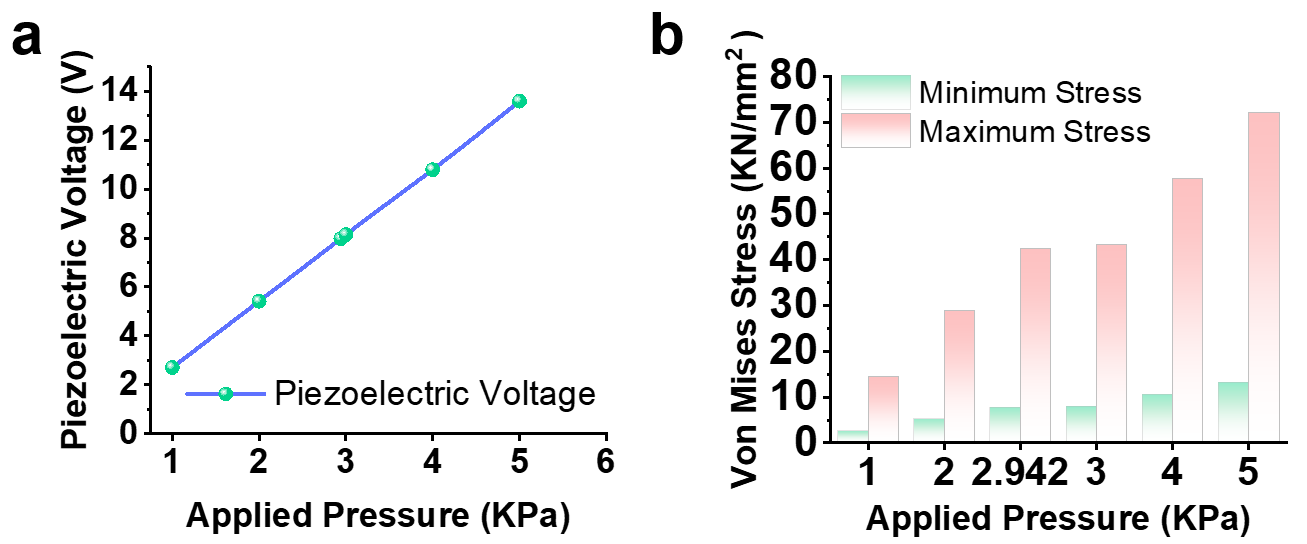


**Supplementary Fig. 21 A comparison of piezoelectric responses under varying pressures. a)** The curve of piezoelectric voltage-applied pressure, **b)** The Von Mises Stress distribution when the applied pressure from 1 Kpa to 5 Kpa.


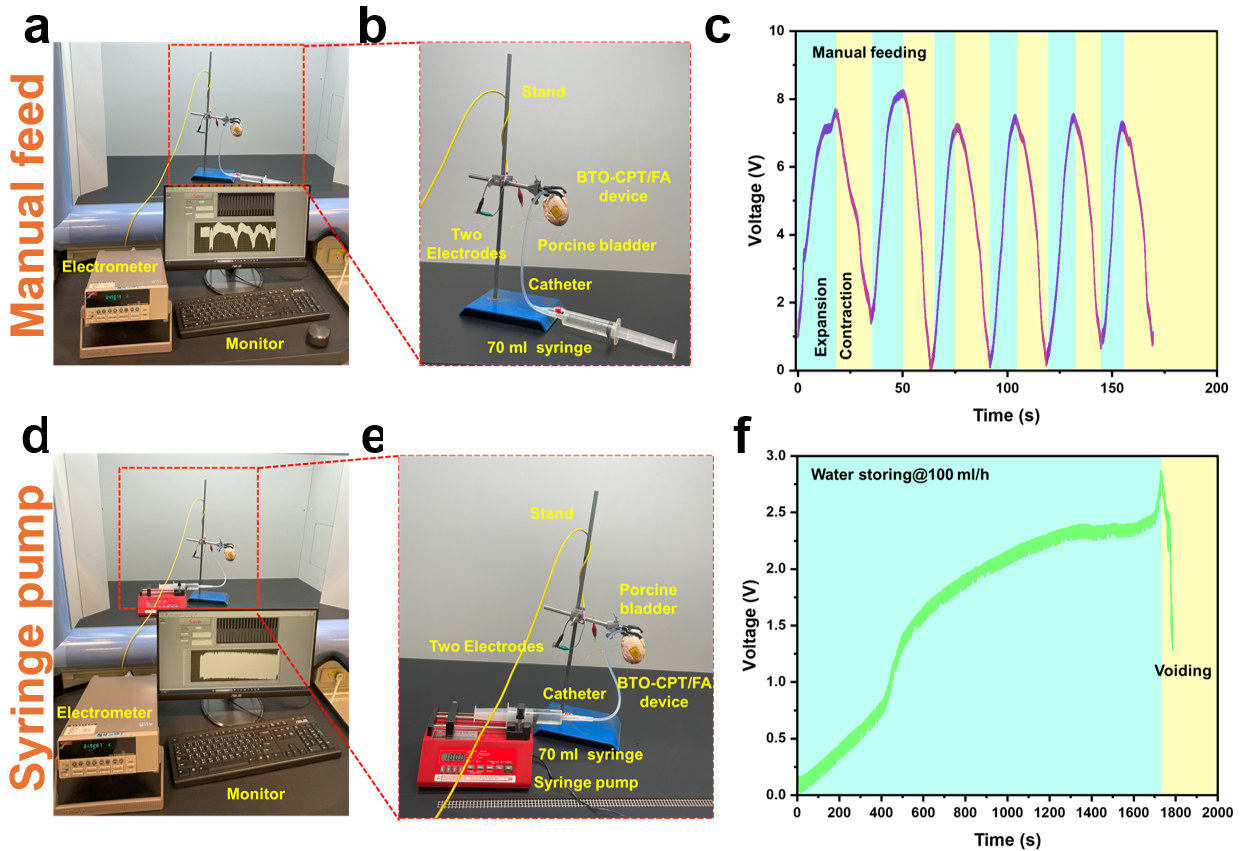


**Supplementary Fig. 22 Experimental setup for evaluating the porcine bladder sensing system under *ex-vivo* conditions. a, b)** Experimental setup simulating the storage and voiding functions of the urinary bladder. **c)** Voltage output variation during the manual injection of deionized (DI) water using a 70 mL syringe. **d, e)** Experimental setup utilizing a syringe pump to maintain a constant DI water flow rate of 100 mL/h. **f)** Corresponding voltage response recorded during the injection of 60 mL of DI water.


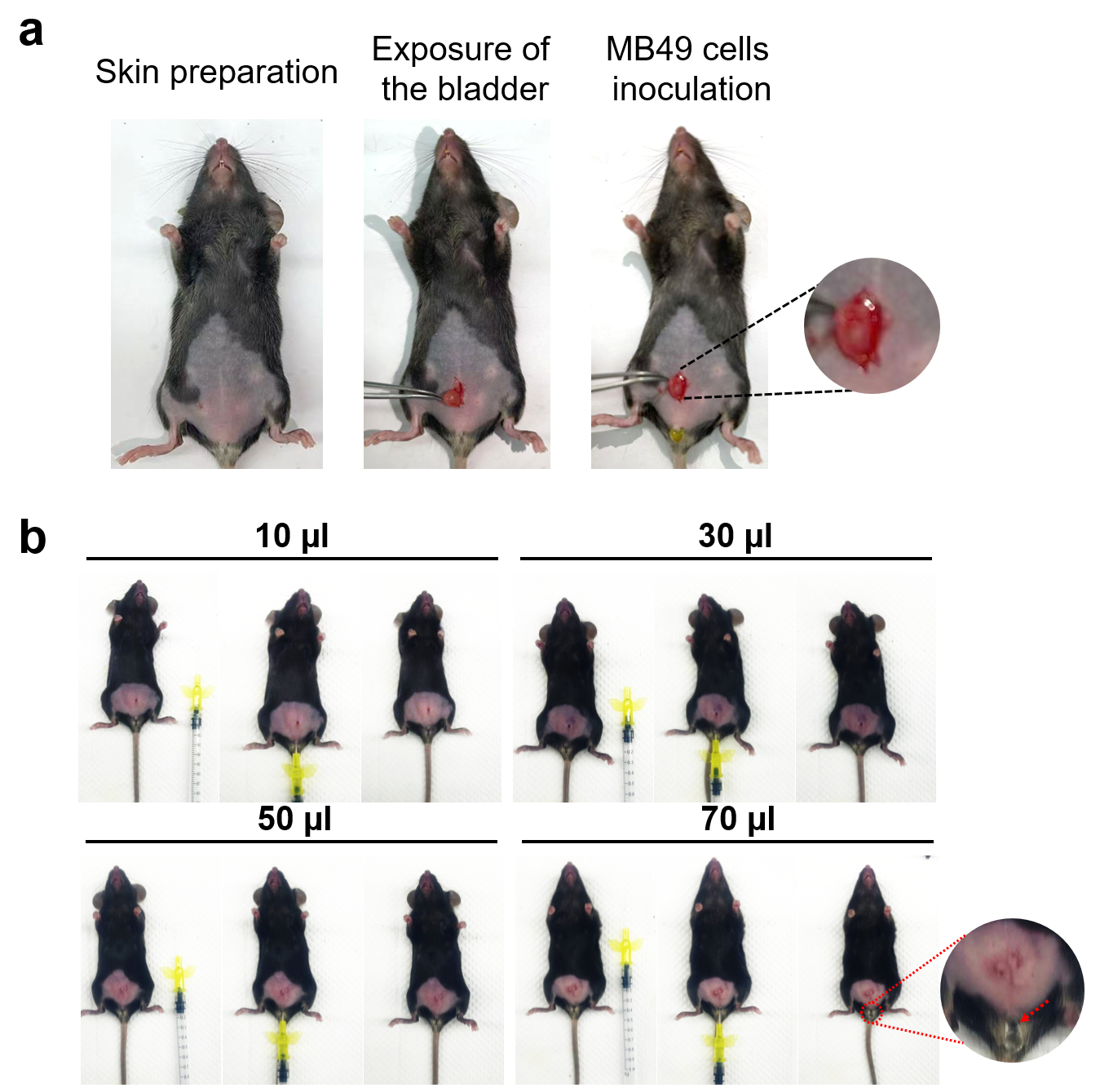


**Supplementary Fig. 23** a) Experimental picture of MB49 cells inoculated within the bladder wall of C57BL/6 mouse. The enlarged image refers to MB49 cells inoculated within the bladder wall. b) The pictures of bladder perfusion with different perfusion volume.


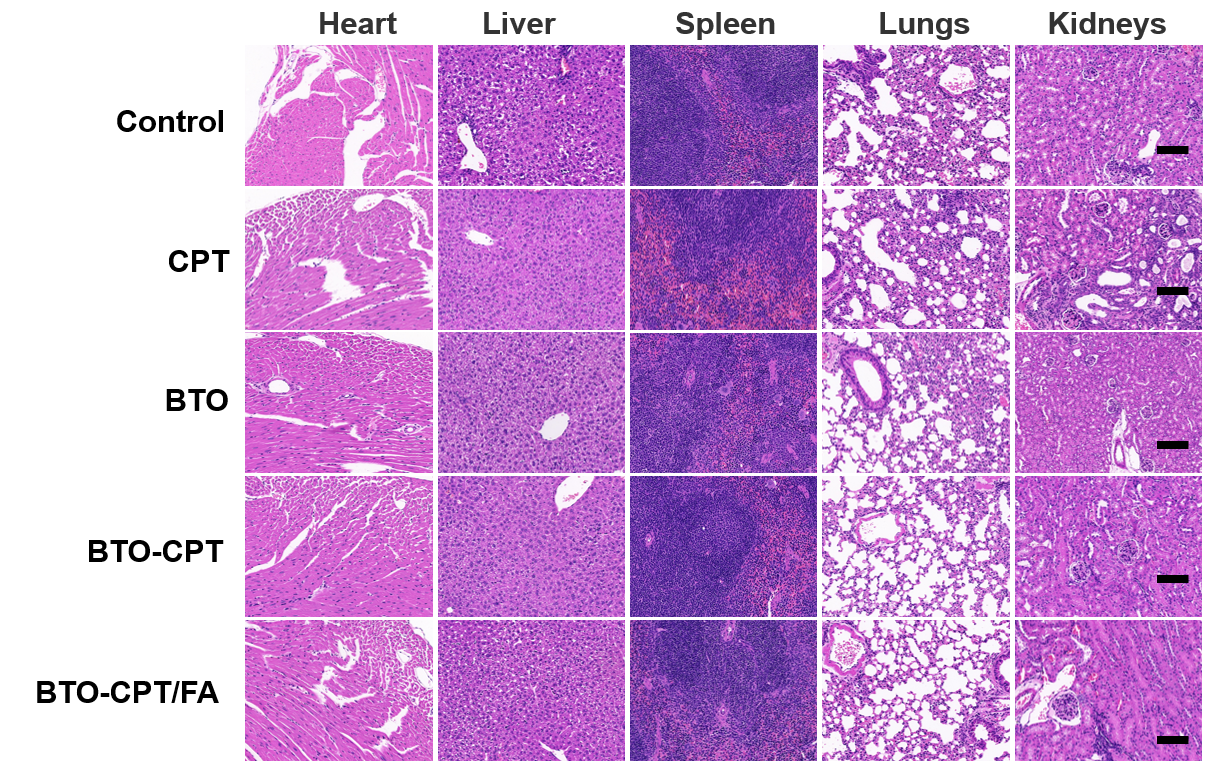


**Supplementary Fig. 24 Evaluation of biosafety in tumor-bearing C57BL/6 mice after treatment with different groups.** H&E staining of the major organs including heart, liver, spleen, lung and kidney collected from different groups after 25 days treatments. scale bar = 100 µm.


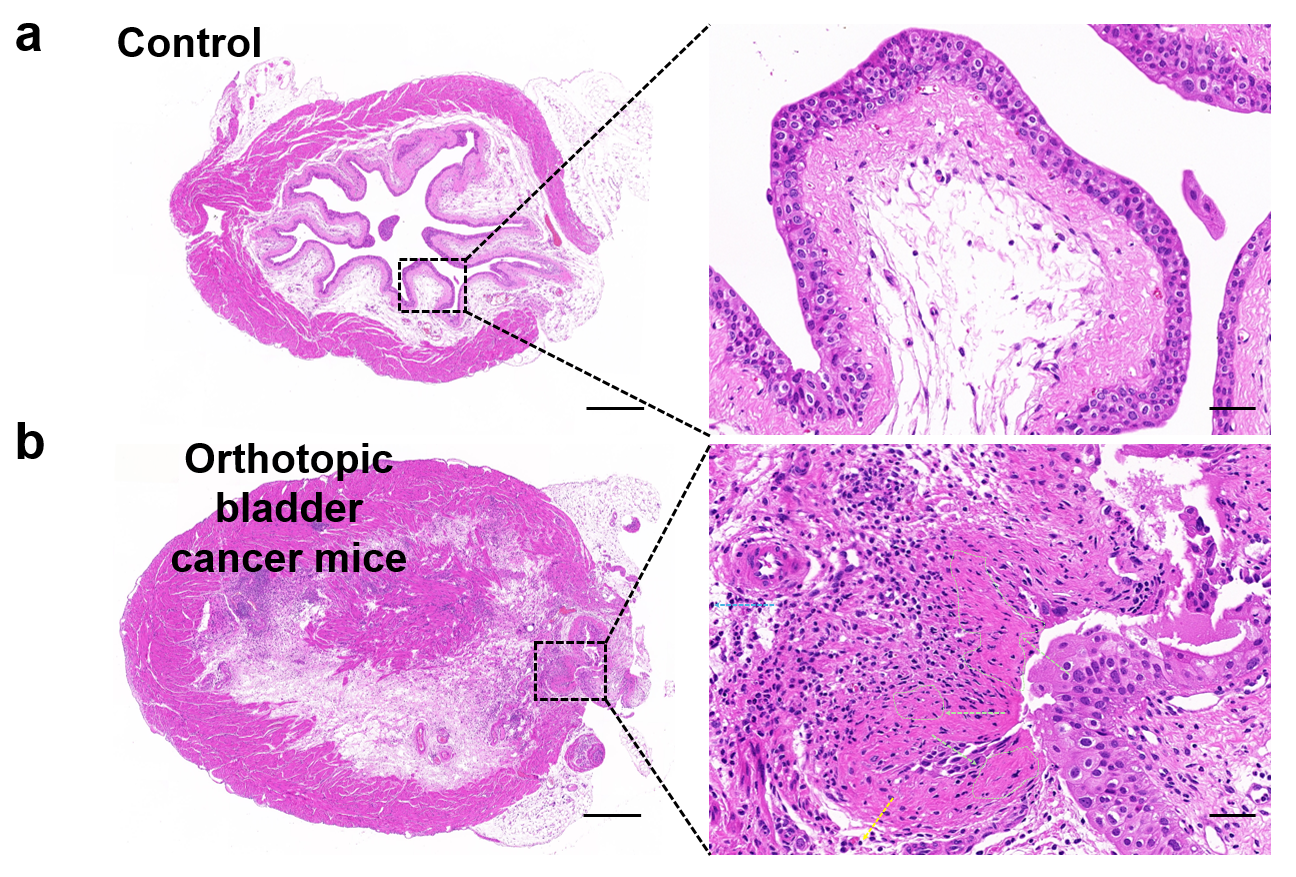


**Supplementary Fig. 25** H&E staining of the bladder collected from different groups after treatment with BTO-CPT/FA. There are cellular debris (green dashed circles), and infiltration of neutrophils (indigo arrows) and lymphocytes (yellow arrows) in the region of orthotopic bladder cancer mice. Scale bar = 500 µm (left), 50 µm (right).


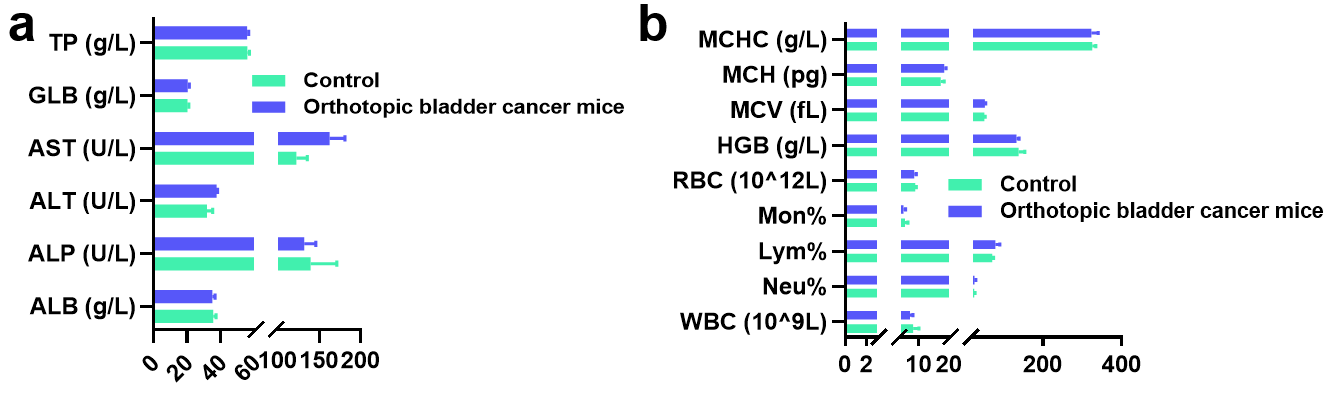


**Supplementary Fig. 26** Analysis of blood-related parameters of healthy mice and orthotopic bladder cancer mice after treatment with BTO-CPT/FA (n = 3).


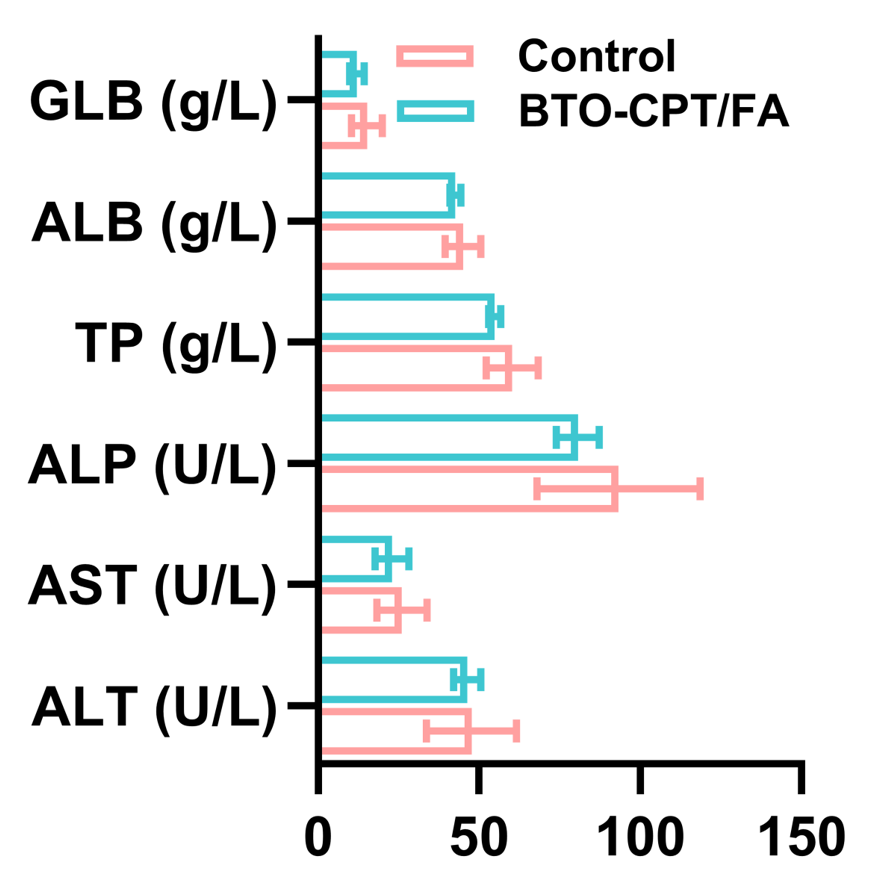


**Supplementary Fig. 27 Analysis of blood chemical parameter in orthotopic bladder cancer rabbit after treatment with BTO-CPT/FA.**


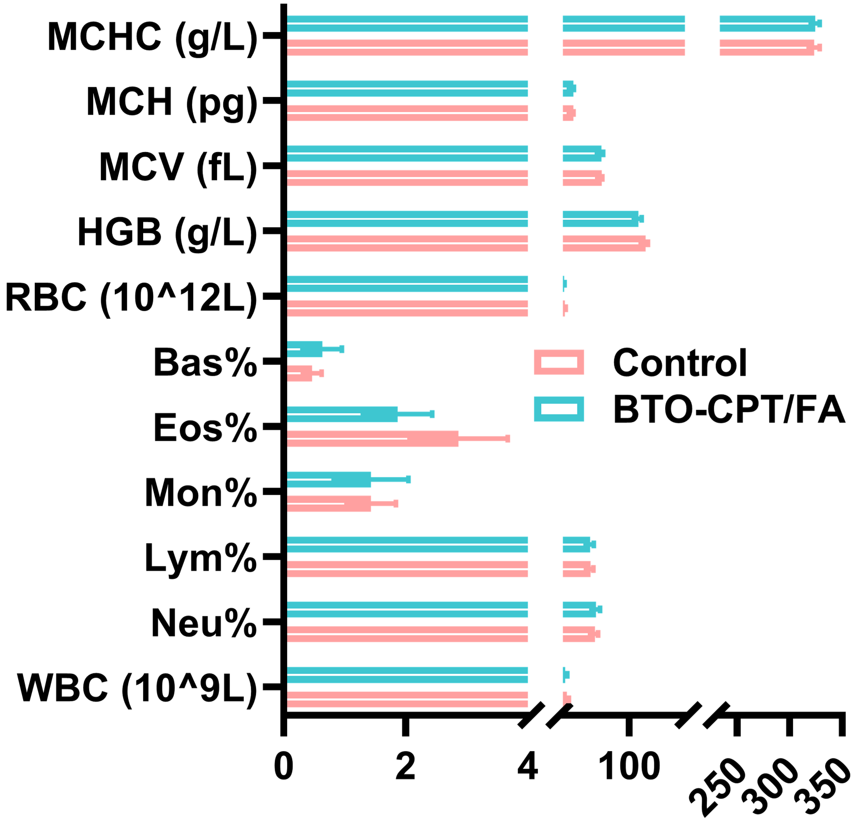


**Supplementary Fig. 28 Analysis of blood-related parameters in orthotopic bladder cancer rabbit after treatment with BTO-CPT/FA.**


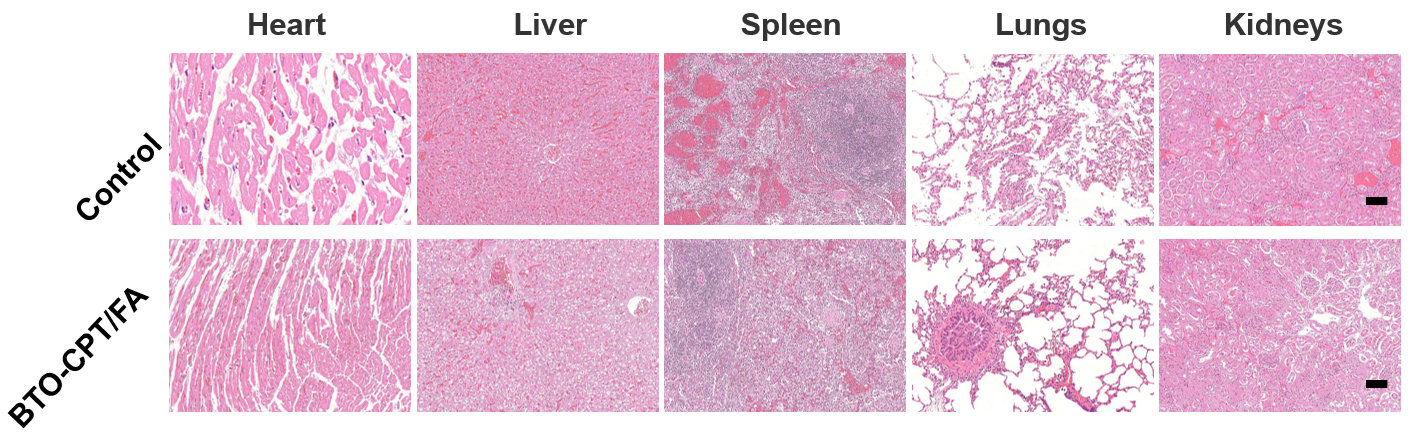


**Supplementary Fig. 29 Evaluation of biosafety in tumor-bearing rabbit after treatment with different groups.** H&E staining of the major organs including heart, liver, spleen, lung and kidney collected from different groups after 45 days treatments. Scale bar = 100 µm.


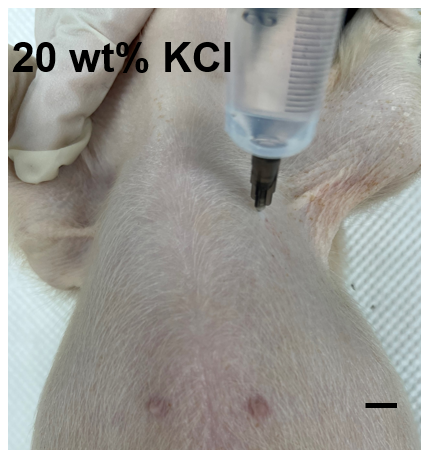


**Supplementary Fig. 30 Mini pigs were executed via intracardiac injection of KCl after the bladder permeability experiment, scale bar = 1 cm.**


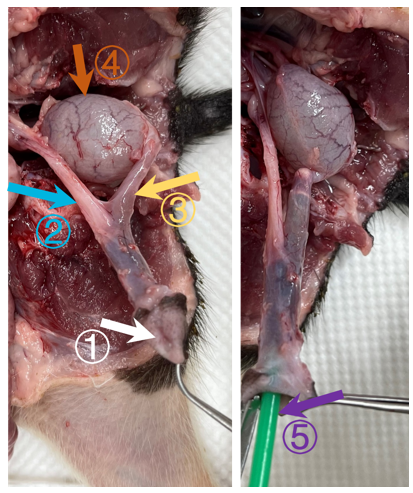


**Supplementary Fig. 31 Anatomical illustration of a mini pig and the specific location refers to vulva (①), cervix (②), urethra (③), bladder (④) and urinary catheter (⑤).**

**Supplementary Table 1** Optimization process in chemical coupling of CPT on BTO

| Number | CPT/mmol | BTO/mg | CPT loading rate/Wt% |
| --- | --- | --- | --- |
| ① | 0.01 | 80 | 5.5 |
| ② | 0.01 | 100 | 6.2 |
| ③ | 0.01 | 120 | 6.5 |

**Supplementary Table 2** Patient sample information

| Patient | Diagnosis | Sex | Age range | Tumor site |
| --- | --- | --- | --- | --- |
| Patient 1 | Bladder cancer (WHO grade G1) | Female | 50-60 | Bladder trigone |
| Patient 2 | Bladder cancer (WHO grade G3) | Female | 60-70 | Bladder trigone |
