# Supplementary material for: Self-driven electrical triggering system activates tunneling nanotube highways to enhance drug delivery in bladder cancer therapy
Source: Nat Commun. 2025 Nov 18;16:10093. doi: 10.1038/s41467-025-65017-2 (PMC12627709; doi:10.1038/s41467-025-65017-2)
Supplement: Supplementary file 2 — Description of Additional Supplementary Files [file 41467_2025_65017_MOESM2_ESM.pdf]

Title: Supplementary Movie. 1
Description: Real-time monitoring of CPT release form BTO-CPT nano-system rely on electrical stimulation in the force of a linear reciprocating motion instrument within seven days.

Title: Supplementary Movie. 2
Description: SPT images of Cy5.5 nanoparticles transported among MB49 cells through TNTs in a series of time intervals, scale bar = 10 μm.

Title: Supplementary Movie. 3
Description: SPT images of BTO-Cy5.5/FA nanoparticles transported among MB49 cells through TNTs in a series of time intervals, scale bar = 10 μm.

Title: Supplementary Movie. 4
Description: SPT images of BTO-Cy5.5/FA nanoparticles＋ultrasound transported among MB49 cells through TNTs in a series of time intervals, scale bar = 10 μm.

Title: Supplementary Movie. 5
Description: FRAP images of free Cy5 nanoparticles in MB49 cells during a series of time intervals, scale bar = 10 μm.

Title: Supplementary Movie. 6
Description: FRAP images of BTO-Cy5/FA nanoparticles in MB49 cells during a series of time intervals, scale bar = 10 μm.

Title: Supplementary Movie. 7
Description: FRAP images of BTO-Cy5/FA nanoparticles＋ultrasound in MB49 cells during a series of time intervals, scale bar = 10 μm.

Title: Supplementary Movie. 8
Description: A device consisting of a PDMS+BTO-CPT/FA piezoelectric layer designed to simulate bladder contraction and expansion in vitro under reciprocating linear movement forces. The device includes a PDMS-BTO piezoelectric layer fixed to a balloon, the balloon itself, a reciprocating linear movement mechanism, and an electrical signal receiving system.

Title: Supplementary Movie. 9
Description: A device featuring a PDMS+BTO-CPT/FA piezoelectric layer, designed to simulate bladder contraction and expansion under reciprocating linear movement forces. The device includes a PDMS-BTO piezoelectric layer fixed to a balloon, a syringe (30 ml) without a needle, a reciprocating linear movement mechanism, and an electrical signal receiving system. The device is shown in operation at first week (left) and second weeks (right).

Title: Supplementary Movie. 10
Description: Demonstration of a device featuring a PDMS+BTO- CPT/FA composite piezoelectric layer, designed to mimic the bladder's fluid storage and voiding condition. Bladder mimicking of the catheter with precise flow control using a syringe pump for realistic expansion and contraction.

Title: Supplementary Movie. 11
Description: Dynamics monitoring the potential of the porcine bladder model in ex-vivo experiment. Forward stroke of the syringe to emulate bladder expansion, conversely, during the backward stroke, bladder contraction.
