# Supplementary material for: Self-driven electrical triggering system activates tunneling nanotube highways to enhance drug delivery in bladder cancer therapy
Source: Nat Commun. 2025 Nov 18;16:10093. doi: 10.1038/s41467-025-65017-2 (PMC12627709; doi:10.1038/s41467-025-65017-2)
Supplement: Supplementary file 14 — Reporting Summary [file 41467_2025_65017_MOESM14_ESM.pdf]

nature portfolio

Corresponding author(s): Yang-Bao Miao

Last updated by author(s): Aug 1, 2025

Reporting Summary

Nature Portfolio wishes to improve the reproducibility of the work that we publish. This form provides structure for consistency and transparency in reporting. For further information on Nature Portfolio policies, see our Editorial Policies and the Editorial Policy Checklist.

Statistics

For all statistical analyses, confirm that the following items are present in the figure legend, table legend, main text, or Methods section.

n/a	Confirmed	
☐	☒	The exact sample size (*n*) for each experimental group/condition, given as a discrete number and unit of measurement
☐	☒	A statement on whether measurements were taken from distinct samples or whether the same sample was measured repeatedly
☐	☒	The statistical test(s) used AND whether they are one- or two-sided<br>*Only common tests should be described solely by name; describe more complex techniques in the Methods section.*
☒	☐	A description of all covariates tested
☒	☐	A description of any assumptions or corrections, such as tests of normality and adjustment for multiple comparisons
☐	☒	A full description of the statistical parameters including central tendency (e.g. means) or other basic estimates (e.g. regression coefficient) AND variation (e.g. standard deviation) or associated estimates of uncertainty (e.g. confidence intervals)
☐	☒	For null hypothesis testing, the test statistic (e.g. *F*, *t*, *r*) with confidence intervals, effect sizes, degrees of freedom and *P* value noted<br>*Give P values as exact values whenever suitable.*
☒	☐	For Bayesian analysis, information on the choice of priors and Markov chain Monte Carlo settings
☒	☐	For hierarchical and complex designs, identification of the appropriate level for tests and full reporting of outcomes
☒	☐	Estimates of effect sizes (e.g. Cohen's *d*, Pearson's *r*), indicating how they were calculated

Our web collection on statistics for biologists contains articles on many of the points above.

Software and code

Policy information about availability of computer code

Data collection	Transmission Electron Microscope (JEM-F2000), Scanning electron microscope (ZEISS-sigma300), nanoparticle size analyzer (Malvern Zetasizer Nano S90), Ultraviolet-Visible Spectrophotometer (UV-3600 Plus), Fourier Transform Infrared Spectrometer (Thermo Fisher iS5), thermogravimetric analysis (HITACHI-STA 200), X-ray Diffractometer (Rigaku Ultima IV), Piezoelectric Force Microscope (MFP-3D),IVIS Lumina II imaging system (Xenogen, USA).
Data analysis	Microsoft Excel and PowerPoint (2016), Graphpad prism (8.0), Image J.

For manuscripts utilizing custom algorithms or software that are central to the research but not yet described in published literature, software must be made available to editors and reviewers. We strongly encourage code deposition in a community repository (e.g. GitHub). See the Nature Portfolio guidelines for submitting code & software for further information.

Data

Policy information about availability of data

All manuscripts must include a data availability statement. This statement should provide the following information, where applicable:

- Accession codes, unique identifiers, or web links for publicly available datasets
- A description of any restrictions on data availability
- For clinical datasets or third party data, please ensure that the statement adheres to our policy

The paper and Supplementary Information contain all data required to assess the study conclusions. Source data are included with this publication.

Research involving human participants, their data, or biological material

Policy information about studies with human participants or human data. See also policy information about sex, gender (identity/presentation), and sexual orientation and race, ethnicity and racism.

Reporting on sex and gender	Female, age range: 50-70.
Reporting on race, ethnicity, or other socially relevant groupings	Although the samples were obtained from patients of the Han nationality, ethnicity was not considered as a variable in the analysis of the samples. Given the limited access to bladder cancer patients samples, conclusions drawn from this research may not account for potential variations attributable to ethnic differences.
Population characteristics	Healthy and tumor tissue samples obtained from 2 patients with different grade bladder cancer were conducted to experiments including H&E staining and live-dead staining.This information of patients are provided in (Supplementary Table 2).
Recruitment	Participants were not specifically recruited for this study, healthy and tumor tissue from patients samples were provided by Department of Oncology, Sichuan Provincial People's Hospital.
Ethics oversight	Approved by the Ethical Review Committees of Sichuan Provincial People's Hospital and the University of Electronic Science and Technology of China (Institutional Animal Care and Use Committee (IACUC) number: 202514).

Note that full information on the approval of the study protocol must also be provided in the manuscript.

Field-specific reporting

Please select the one below that is the best fit for your research. If you are not sure, read the appropriate sections before making your selection.

☒ Life sciences ☐ Behavioural & social sciences ☐ Ecological, evolutionary & environmental sciences

For a reference copy of the document with all sections, see nature.com/documents/nr-reporting-summary-flat.pdf

Life sciences study design

All studies must disclose on these points even when the disclosure is negative.

Sample size	Sample size was calculated using Graphpad prism (8.0) for a two-tailed t-test, one-way ANOVA based on pilot data (n≥3/group).
Data exclusions	No data were excluded from the analyses.
Replication	All attempts at replication were successful.
Randomization	Randomized groups based on weight/age in animal experiments.
Blinding	Tumor volume measurements, fluorescence imaging and bioluminescence were performed by independent researchers blinded to treatment-group assignments.

Reporting for specific materials, systems and methods

We require information from authors about some types of materials, experimental systems and methods used in many studies. Here, indicate whether each material, system or method listed is relevant to your study. If you are not sure if a list item applies to your research, read the appropriate section before selecting a response.

Materials & experimental systems

n/a	Involved in the study	
☐	☒	Antibodies
☐	☒	Eukaryotic cell lines
☒	☐	Palaeontology and archaeology
☐	☒	Animals and other organisms
☐	☒	Clinical data
☒	☐	Dual use research of concern
☒	☐	Plants

Methods

n/a	Involved in the study	
☒	☐	ChIP-seq
☒	☐	Flow cytometry
☒	☐	MRI-based neuroimaging

Antibodies

Antibodies used	CD31 Monoclonal Antibody, FITC (Mouse monoclonal, ThermoFisher scientific, RM5201, Clone: 390, RRID: AB_10373983, for immunofluorescence).
Validation	All antibodies were validated by the supplier, with each lot undergoing quality control testing. These antibodies were used according to the manufacturers' recommended applications without further independent validation.

Eukaryotic cell lines

Policy information about cell lines and Sex and Gender in Research

Cell line source(s)	The MB49 cells (Mouse bladder carcinoma cells), MB49-Luc cells (Luciferase-expressing MB49), SV-HUC-1 (a type of bladder epithelial cell) and Vx2 (Rabbit squamous cell carcinoma cells) were purchased from the Cell Bank of the Chinese Academy of Sciences (Shanghai, China).
Authentication	These cell lines were morphologically confirmed.
Mycoplasma contamination	All cell lines tested negative for mycoplasma contamination.
Commonly misidentified lines (See ICLAC register)	The cell lines utilized were confirmed to be authentic, with none appearing on the ICLAC register of frequently misidentified cell lines.

Animals and other research organisms

Policy information about studies involving animals; ARRIVE guidelines recommended for reporting animal research, and Sex and Gender in Research

Laboratory animals	C57BL/6 mice (female, 5~7 weeks old) were purchased from SPF Biotechnology (Beijing, China), New Zealand rabbits (female, 2.5~3 months old) and minipigs (female, 4~5 weeks old) were purchased from Chongqing Tengxin Biotechnology Co., Ltd. Animal care was provided in an SPF-certified vivarium where subjects had continuous access to feed and water. Standard housing conditions (22°C-25°C, 40-60% humidity) with controlled lighting (12h light/12h dark) were maintained for all experimental groups, with 3-4 animals housed together.
Wild animals	Did not involve wild animals.
Reporting on sex	Female.
Field-collected samples	No field-collected samples were involved in this paper.
Ethics oversight	All animal experiments were approved by the Ethical Review Committees of Sichuan Provincial People's Hospital and the University of Electronic Science and Technology of China (Institutional Animal Care and Use Committee (IACUC) number: 202514), following the standards set by the National Institutes of Health Guide for the Care and Use of Laboratory Animals.

Note that full information on the approval of the study protocol must also be provided in the manuscript.

Clinical data

Policy information about clinical studies

All manuscripts should comply with the ICMJE guidelines for publication of clinical research and a completed CONSORT checklist must be included with all submissions.

Clinical trial registration	*Provide the trial registration number from ClinicalTrials.gov or an equivalent agency.*
Study protocol	*Note where the full trial protocol can be accessed OR if not available, explain why.*
Data collection	*Describe the settings and locales of data collection, noting the time periods of recruitment and data collection.*
Outcomes	*Describe how you pre-defined primary and secondary outcome measures and how you assessed these measures.*

Plants

Seed stocks	none
Novel plant genotypes	none
Authentication	none
